# Comparative physiology of glomerular filtration rate by plasma clearance of exogenous creatinine and exo-iohexol in six different avian species

**DOI:** 10.1038/s41598-019-56096-5

**Published:** 2019-12-23

**Authors:** Elke Gasthuys, Andrés Montesinos, Nele Caekebeke, Mathias Devreese, Siegrid De Baere, Maria Ardiaca, Dominique Paepe, Siska Croubels, Gunther Antonissen

**Affiliations:** 1 Department of Pharmacology, Toxicology and Biochemistry, Faculty of Veterinary Medicine, Ghent University, Salisburylaan 133, 9820 Merelbeke, Belgium; 2 Centro Veterinario los Sauces, Calle Santa Engracia 63, 28010 Madrid, Spain; 3 Department of Pathology, Bacteriology and Avian Diseases, Faculty of Veterinary Medicine, Ghent University, Salisburylaan 133, 9820 Merelbeke, Belgium; 4 Small Animal Department, Faculty of Veterinary Medicine, Ghent University, Salisburylaan 133, 9820 Merelbeke, Belgium

**Keywords:** Glomerulus, Zoology

## Abstract

Early diagnosis of kidney diseases in avian species is limited. Endogenous markers currently used in avian practice are not sensitive enough to identify early kidney failure. Consequently, alternative markers should be evaluated. To be able to evaluate these alternative markers, an accurate marker to estimate the GFR should be validated. This study determined the GFR, measured as clearance of exogenous creatinine and exo-iohexol, in six different bird species, i.e. broiler chickens, laying chickens, turkeys, Muscovy ducks, pigeons and African grey parrots (4♀/4♂). To be able to compare the six bird species, normalization to bodyweight (BW) of the GFR was performed, after a good correlation between BW and kidney weight was demonstrated (R² = 0.9836). Clearance of exo-iohexol normalized to BW (mL/min/kg) was determined in all bird species, i.e. 3.09 in broiler chickens; 2.57 in laying chickens; 1.94 in turkeys; 1.29 in pigeons; 2.60 in ducks and 1.11 in parrots. However, these results differed significantly with the clearance of exogenous creatinine: 8.41 in broiler chickens; 9.33 in laying chickens; 5.62 in turkeys; 14.97 in pigeons; 17.59 in ducks and 25.56 in parrots 25.56. Iohexol is preferred to measure the GFR, since it is not prone to tubular reabsorption nor secretion.

## Introduction

Renal disease in avian species is frequently encountered in clinical practice (approximately 30% of all disease conditions) and can be caused by a large number of disease processes, such as dehydration, hypovitaminosis A, heavy metal toxicity, mycotoxic nephropathy (ochratoxin A), infectious diseases, renal lipidosis, amyloidosis, and renal carcinoma^1,2^. Kidneys are dynamic organs which are directly or indirectly associated with multiple body systems. As a result, renal disorders can lead to or be the result of multiple other disease processes^2^. The *ante mortem* diagnosis of renal disorders in birds is challenging as pathognomonic signs are rare^3^. Furthermore, early diagnosis of kidney diseases in avian species is limited because most of the commonly used markers, i.e. uric acid, are not sensitive enough to detect renal failure and urine collection is difficult to perform under clinical conditions^2^.

In humans and mammalian species, assessment of the glomerular filtration rate (GFR) is considered as the gold standard to evaluate the kidney function^4,5^. Accurate assessment of the GFR in human and veterinary research is essential for early detection of renal failure. Extrapolation of the GFR from mammals to birds is not recommended, since the gross external morphology of the avian kidneys differs from mammalian kidneys or the snake kidneys, but closely resembles the external morphology of chelonian and saurian reptiles kidneys. Two types of nephrons can be distinguished in the avian kidney, namely cortical or reptilian-type (70–90%, simple nephrons without loops of Henle) and medullary or mammalian-type nephrons (10–30%, complex nephrons with loops of Henle)^6^. The avian kidney is not comparable to the mammalian kidney and the mammalian-type nephrons are less prone to a decrease in GFR in comparison with the reptilian-type nephrons. Therefore, the assessment of the GFR should be specifically evaluated in avian species^5^.

To date, information about the GFR in different avian species is rather scarce. Only a couple of scientific reports mention the determination of the GFR in specific bird species. Moreover, to the authors’ knowledge, no research has been done about the correlation of the GFR among different avian species, whereby it is not known if the GFR obtained in one avian species might be extrapolated to other avian species. Skadhauge and Schmidt-Nielsen^7^, Braun and Dantzler^8^, Wideman *et al*.^9^ and Gerson and Guglielmo^10^ measured the GFR, based on inulin administration, in domestic fowls (*Gallus gallus dom.*, bodyweight (BW): 1–3 kg, GFR: 1.73–2.12 mL/min/kg), desert quails (*Lophortyx gambelii*, BW: 140–160 g, GFR: 0.88 mL/min/kg), broiler chickens (*Gallus gallus dom.*, different ages: 1, 3, 5, 9, 12, 21 and 30 weeks, GFR: 2.58–3.15 mL/min/kg) and Swainson’s thrushes (*Catharus ustulatus*, BW: 30 g, GFR: 16.82 mL/h), respectively. Goldstein and Rothschild^11^ assessed the GFR, based on polyethylene glycol administration, in captive and wild song sparrows (BW: 18.4 ± 2 g, GFR: 5.3–9.0 mL/h). Scope *et al*.^5^ determined the GFR by exogenous creatinine administration in racing pigeons (*Columba livia*, BW: 491 ± 42 g, GFR IV: 6.30 mL/min/kg, GFR IM: 6.44 mL/min/kg). The authors concluded that both intravenous (IV) as well as intramuscular (IM) administration of exogenous creatinine accurately predicted the GFR in pigeons.

Since the GFR cannot be measured directly, assessment of the GFR is performed using clearance markers. To date, the frequently used endogenous biomarkers in veterinary practice, namely serum concentrations of creatinine, uric acid or urea and urine specific gravity, are not sensitive enough to detect early kidney dysfunction^2^. Consequently, alternative endogenous biomarkers, such as cystatin C, symmetric demethylarginine or N-acetyl-β-D-glucosaminidase, should be evaluated. In order to evaluate if these markers are sensitive enough to detect early renal failure, a reference standard clearance marker to accurately predict the GFR in avian species should be validated. The ideal clearance marker for GFR determination should be freely filtered by the glomerulus, safe, inexpensive and not subjected to tubular reabsorption, tubular secretion nor plasma protein binding^12^. Renal clearance of inulin has been widely acknowledged as the gold standard in measuring the GFR in both human and veterinary research. However, determination of inulin clearance is costly, time-consuming and difficult to perform since IV infusion and timed urine collections are required. Therefore, alternative clearance markers such as the non-ionic contrast agent iohexol and exogenous creatinine have been proposed. Determination of iohexol and creatinine clearance are easily performed, since they do not require urine collection (difficult to perform in avian species, because urine is excreted together with faeces) or specialized equipment (in contrast with for example radionuclides). Creatinine and iohexol have been used frequently in human and veterinary (cat and dog) nephrology^4,13,14^, but not in avian medicine.

The aim of the present study was to assess a reference standard clearance marker, i.e. exogenous exo-iohexol and creatinine, to estimate the GFR in six different avian species (broiler chickens, laying chickens, turkeys, Muscovy ducks, pigeons and African grey parrots (AGP)). Moreover, the obtained GFR estimations were compared among and correlated between different avian species using both BW and kidney weight (KW) as scaling factors.

## Results

**Figure 1:**
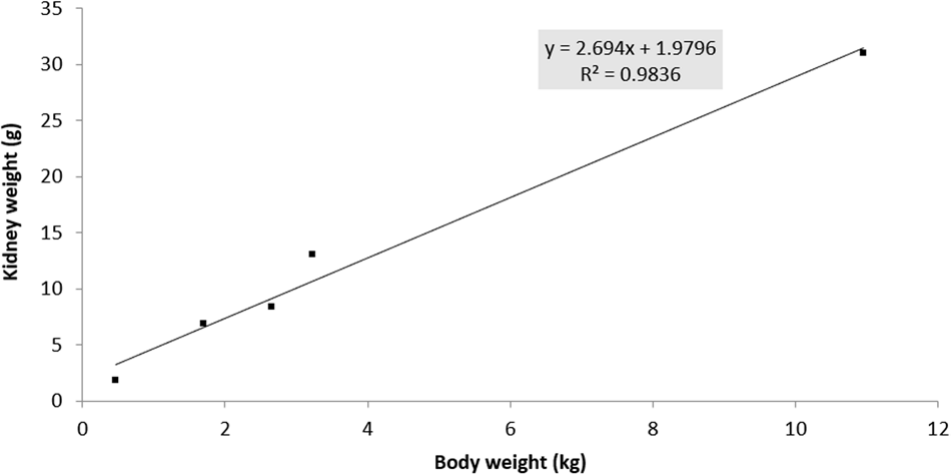
Correlation between mean kidney weight and mean body weight in five different avian species (broiler chicken, laying chicken, turkey, duck, and pigeon). Kidneys of the African Grey parrots were not collected during this study.

**Figure 2:**
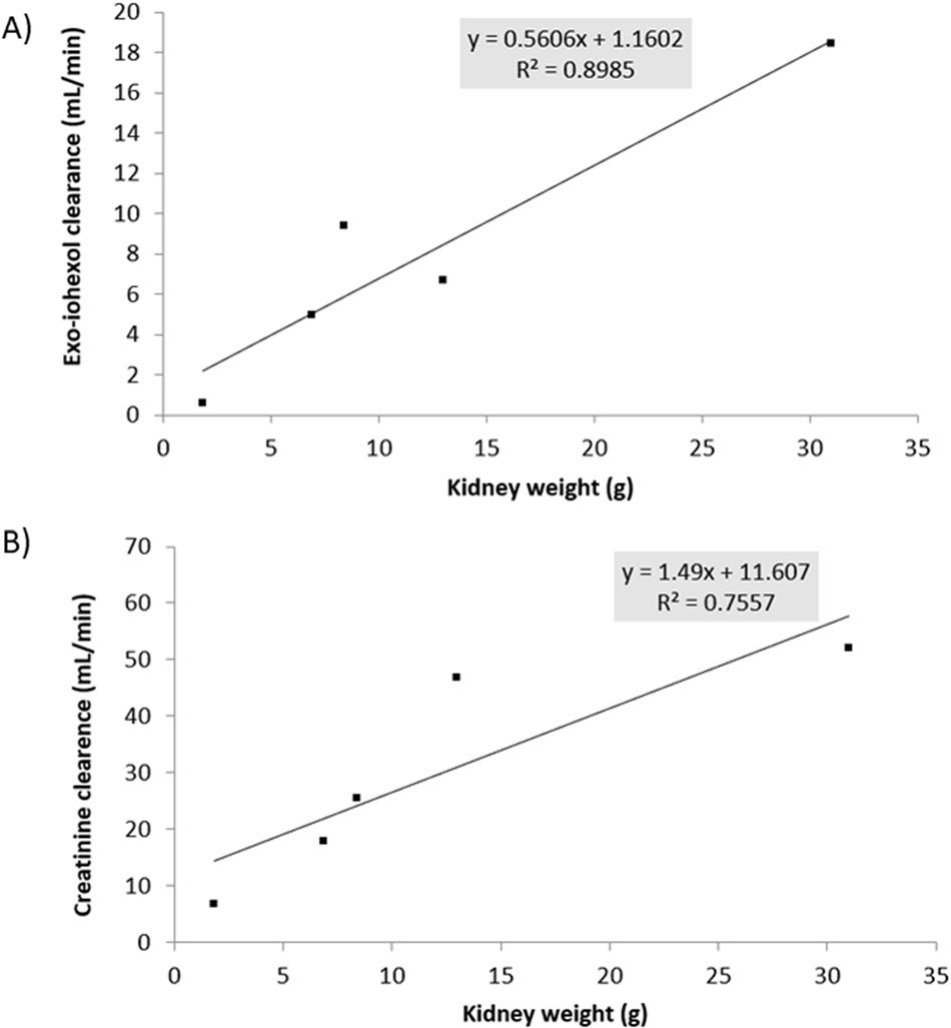
Allometric scaling of kidney weight (g) and exo-iohexol (**A**), and creatinine (**B**) clearance (mL/min) for five different avian species (broiler chicken, laying chicken, turkey, duck, and pigeon).

**Figure 3:**
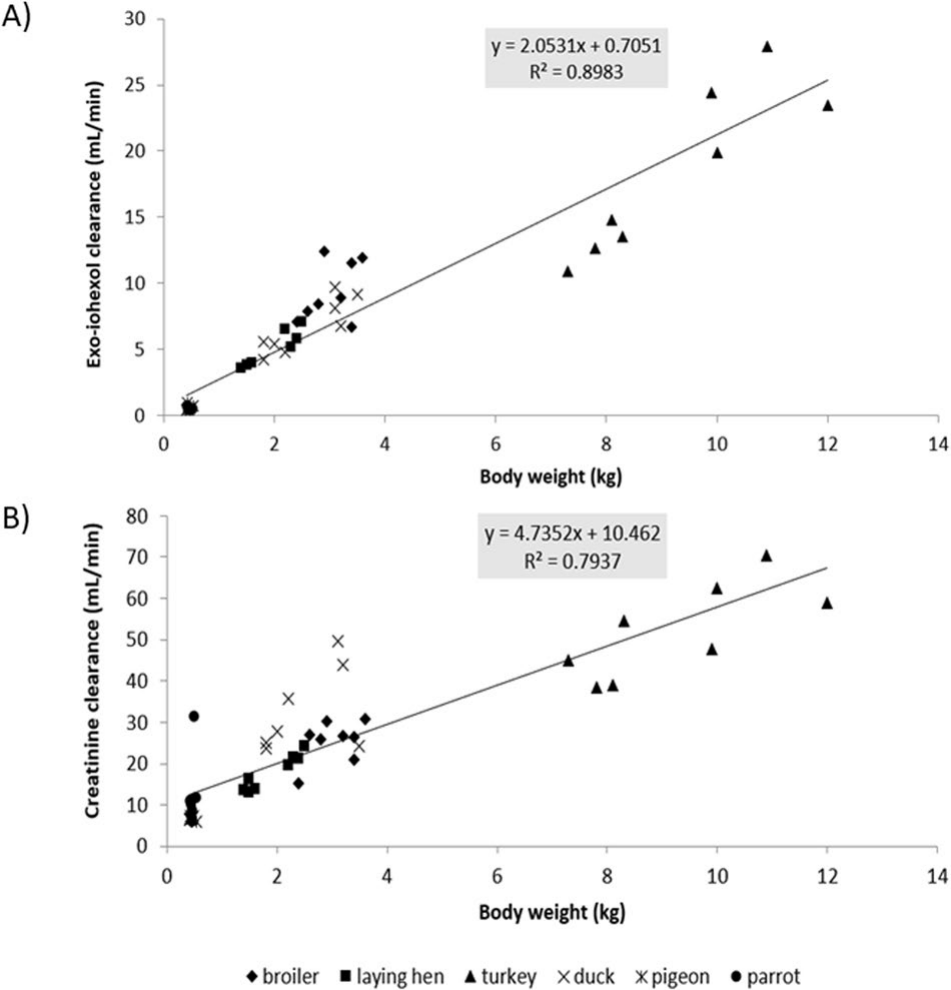
Allometric scaling of body weight (kg) and exo-iohexol (**A**), and creatinine (**B**) clearance (mL/min) for six different avian species (broiler chicken, laying chicken, turkey, pigeon, duck, and parrot).

All birds survived the experiment without any complications. One pigeon was excluded from the study due to technical issues during blood sampling. Table 1 summarizes the descriptive statistics of the different bird species included in the study. In the presented study, a linear correlation (R² = 0.9836) between BW and KW was observed for the five bird species (broiler chicken, laying chicken, turkey, duck and pigeon) for which KW was available (Fig. 1). Hence, both KW and BW can be interchangeably used as scaling factors to normalize the GFR in birds. This observation was also reflected in Fig. 2A, where allometric scaling demonstrated a good correlation between KW of the five different avian species and clearance of exo-iohexol (R² = 0.8985). The correlation between KW of the avian species and clearance of creatinine is less in comparison to the clearance of exo-iohexol (R² = 0.7557, Fig. 2B). The mean GFR values indexed to BW of the different markers (mean ± SD) are presented in Table 2. Significant differences between the mean renal clearance of creatinine and iohexol were observed for all bird species. When comparing the different bird species within each technique, a significant difference (p < 0.05) between parrots and three other avian species, namely broiler chickens, laying chickens and turkeys, was observed for all techniques. No other significant differences between the GFR values measured with exogenous creatinine were noted. The GFR values measured with iohexol significantly differed (p < 0.05) between broiler chickens and turkeys, broiler chickens and pigeons, laying chickens and pigeons, pigeons and ducks, ducks and parrots, respectively. No significant sex differences were observed. Allometric scaling clearly demonstrated a correlation between BW of the different avian species and clearance of exo-iohexol, but less for creatinine (R² = 0.8983 and 0.7937, respectively) (Fig. 3A,B).

**Table 1:** Descriptive statistics of the different bird species. Values are mean ± SD.

Species	Gender	Age	Number	Bodyweight (kg)	Kidney weight (g)
Broiler chicken	♂	6 weeks	4	3.40 ± 0.16	9.75 ± 4.43
	♀	6 weeks	4	2.68 ± 0.22	7.00 ± 0.82
Laying chicken	♂	18 weeks	4	2.35 ± 0.13	7.00 ± 1.41
	♀	18 weeks	4	1.50 ± 0.08	6.74 ± 1.89
Turkey	♂	13 weeks	4	10.7 ± 0.98	33.50 ± 2.38
	♀	13 weeks	4	7.88 ± 0.43	28.50 ± 4.43
Pigeon	♂	6–12 months	4	0.48 ± 0.04	1.62 ± 0.20
	♀	6–12 months	3	0.42 ± 0.008	2.04 ± 0.17
Duck	♂	6 months	4	3.23 ± 0.19	15.30 ± 3.14
	♀	6 months	4	1.95 ± 0.19	6.00 ± 0.00
Parrot	♂	4–20 years	4	0.49 ± 0.05	—
	♀	4–20 years	4	0.45 ± 0.01	—

**Table 2:** Glomerular filtration rate measurements (GFR (mL/min/kg), mean ± SD) using creatinine and exo-iohexol in six different avian species (♂/♀). The GFR is indexed to bodyweight.

Species	Creatinine	Exo-iohexol
Broiler chicken	8.41 ± 1.63^a^	3.09 ± 0.65^c^
Laying chicken	9.33 ± 0.73^a^	2.57 ± 0.23^cd^
Turkey	5.62 ± 0.81^a^	1.94 ± 0.39^d^
Pigeon	14.97 ± 1.78^ab^	1.29 ± 0.47^ef^
Duck	17.59 ± 12.20^ab^	2.60 ± 0.38^c^
Parrot	25.56 ± 15.21^b^	1.11 ± 0.34^f^

Results with a different alphabetical character superscript are considered significantly different (p < 0.05) between the different bird species within one technique. For each bird species, the mean renal clearance of creatinine differt significantly with the mean renal clearence of iohexol.

## Discussion

The current study evaluated clearance markers to estimate the GFR in six different avian species. Clearance of exo-iohexol normalized to BW (mL/min/kg) was determined in all bird species, which differed significantly with the clearance of exogenous creatinine. In birds, a reduction of the GFR can be attributed to renal disease or a normal physiologic response to dehydration. When dehydration occurs, the decrease in GFR is intermittent, due to the release of arginine vasotocin which causes vasoconstriction of the afferent arteriole of the reptilian-type nephrons and inhibition of filtration in these nephrons^15^. Since the GFR cannot be measured directly, it is estimated based on the clearance of an endogenous or exogenous marker^4^. In contrast to mammals, in avian species, endogenous creatinine cannot be applied as marker because physiological creatinine concentrations are below the limit of quantification (LOQ) of frequently used field to lab-assays^16,17^. Therefore, alternative clearance markers should be used to measure GFR in birds. On the other hand, Scope *et al*.^5^ demonstrated that IV or IM administration of exogenous creatinine can be useful for assessing renal creatinine excretion in pigeons, but did not compare the obtained results with a gold standard such as inulin or iohexol. In this study in all species, the clearance of creatinine was significantly higher than the clearance of exo-iohexol. This was similar to the results obtained in healthy and hyperthyroid cats, where significant differences between the mean GFR obtained by creatinine and iohexol clearance were observed^14,18^. The avian renal tubules might secrete creatinine when plasma levels are elevated and reabsorb creatinine when plasma levels are normal, whereby plasma concentration levels of creatinine might be confounded^19^. In the future, iohexol can be used as reference standard to evaluate potential biomarkers of early renal dysfunction detection in birds.

Sturkie^20^ mentioned the mean inulin clearance values of chickens (0.60–3.00 mL/min/kg), ducks (2.5 mL/min/kg) and doves (2.6 mL/min/kg). These values are in line with the results reported for exo-iohexol in the current manuscript (chickens:2.57–3.09 mL/min/kg, ducks: 2.60 mL/min/kg, doves: 1.29 mL/min/kg). The results are however not in line with our results observed when administering exogenous creatinine. The GFR values obtained using exo-iohexol in broiler and laying chicken were comparable with the results obtained by Radin *et al*.^21^ and Wideman *et al*.^9^, who determined the clearance of inulin in chickens (3.12 ± 0.43 mL/min/kg and 2.81 ± 0.35 mL/min/kg, respectively). When comparing the creatinine clearance found in this study and the values obtained by Scope *et al*.^5^ in pigeons (6.30 mL/min/kg), the values in the current study (20.90 mL/min/kg) were remarkably higher than those of Scope *et al*.^5^. The values obtained using exo- iohexol obtained in this study (2.51 and 1.14 mL/min/kg, respectively), however, were apparently lower. Roberts *et al*.^22^ demonstrated variability of the GFR values within the same bird species, which might explain the differences observed in the study of Scope *et al*.^5^ and the current study. Moreover, studies in cats and dogs have shown that GFR values widely differ with different techniques (e.g. different clearance markers, different time points, etc.) and different GFR calculation models (e.g. one-compartment versus two-compartment versus non-compartment pharmacokinetic models)^14,23^.

In the current study, the plasma concentration-time curve of iohexol assumes a double-exponential decay of the plasma concentrations, representing a two-compartmental model. In most studies, iohexol clearance is determined using a two-compartmental model approach, necessitating multiple early and late blood samplings^24^. In clinical practice however, taking multiple blood samples is cumbersome for the patients. Therefore, alternative single- and dual-plasma sampling approaches were compared with the traditional urinary clearance or multiple-samples plasma clearance approaches. Zhang *et al*. determined if these new approaches could accurately predict iohexol clearances in 170 humans (46 healthy volunteers, 124 chronic kidney disease). The authors concluded that the one-point sampling approach could be applied in patients with a GFR above 60 mL/min/1.73 m², but could not be applied in patients with a GFR below 60 mL/min/1.73 m². A prolonged sampling time is required in the latter patient group^25^. The latter was confirmed in children by Tøndel *et al*. (single: >30 mL/min/1.73 m²; double: <30 mL/min/1.73 m²)^26^. In future studies, the use of single- or double-plasma sampling approaches could be evaluated in avian species as well, whereby a mathematical correction for the first fast exponential curve based on the slope-intercept method proposed by Brochner-Mortensen could be incorporated^27^. Also the use of a one, versus two versus three compartment model for determining plasma iohexol clearance is topic for debate. As mentioned above, most studies apply a two-compartmental model based on the Schwartz approach^4^, however Taubert *et al*. proposed a three-compartmental model which markedly improved iohexol clearance estimation. Further studies are still required to confirm the aptness of this model^28^.

Since the GFR represents the clearance of a substance by glomerular filtration, scaling of the GFR to a standard measure of body size should be performed to be able to compare GFR values among different avian species. Ideally, the GFR should be scaled using KW, but the availability of KW is clinically not applicable^29^. Therefore, readily available alternative scaling factors, such as BW, body surface area and extracellular fluid volume are frequently used both in human as in veterinary clinical practice. In veterinary clinical practice, BW is the reference to normalize the GFR (i.e. cats and dogs). Also in the current study, a linear correlation (R² = 0.9836) between BW and KW was observed in the different avian species, rendering BW a suitable scaling factor to compare the GFR among these species. In the current study, a clear association between the bird’s BW and the clearance of exo-iohexol was demonstrated by allometric scaling (R² = 0.9138 and 0.8983, respectively). Birds with lower BW, also have lower GFR values (mL/min). This correlation was less pronounced using exogenous creatinine (R² = 0.7937).

Determination of the GFR in avian species is difficult to perform, which brought a number of limitations to the study design:

- Determination of endogenous creatinine was not possible, since the basal values of creatinine are below the LOQ of the enzymatic assay. In the current manuscript it was therefore opted to administer exogenous creatinine to the birds.
- The AGP needed to be anaesthetized in order to be able to administer iohexol and creatinine correctly. Anaesthetics might lower the GFR, however in the current study design anaesthesia was limited in time and iohexol and creatinine were administered after anaesthetic recovery, minimalizing the influence on the GFR.
- Urine collection is difficult to perform in avian species, by which the most direct approach to calculate the GFR, namely urine concentration*urine flow/plasma concentration, is difficult to apply in these species. Therefore, alternative approaches based on plasma concentrations were evaluated in this manuscript circumventing urine collection. In the future, the alternative approaches should be validated with the direct approach, whereby suitable urine collection techniques should be applied.
- Collecting larger volumes of blood in smaller avian species (i.e. AGP and pigeons) is ethically not possible. In the current manuscript it was opted to lower the sampling volume in these avian species. In even smaller avian species, sparse sampling combined with population-PK analysis or dry-blood spots could be suitable alternatives to circumvent rich/large blood sampling.
- Exogenous iohexol is preferred to creatinine to measure the GFR in avian species. This marker however cannot be directly used in clinical practice, since a HPLC-UV technique is often not available. Therefore, iohexol should be rather used in research to evaluate the potential of other renal function markers which could easily be assessed in clinical practice.

In conclusion, the current study determined the GFR in six different avian species. The values obtained with exo-iohexol were comparable, but significantly differed with the clearance of exogenous administered creatinine. Iohexol is preferred to creatinine to measure the GFR in avian species, since it is not prone to tubular reabsorption nor secretion. Iohexol can be used to identify potential biomarkers of early renal dysfunction in avian species.

## Materials and Methods

### Animals

The current study was in accordance with the national^30^ and European legislation^31^ for care and use of laboratory animals and was conducted with consent of the ethical committee of the Faculties of Veterinary Medicine and Bioscience Engineering of Ghent University (EC2015/155; approval: January 18^th^, 2016) and Comité ético de bienestar animal de GREFA (15/0005; approval: October 30^th^, 2015). The experiments were carried out in clinically healthy broiler chickens (Ross 308, *Gallus gallus*), laying chickens (Lohmann Brown-Lite, *Gallus gallus dom.*), turkeys (Hybrid Converter, *Meleagris gallopavo*), Muscovy ducks (*Cairina moschata*), pigeons (racing pigeons, *Columba livia* forma domestica) and AGP (*Psittacus erithacus erithacus*). The study was conducted with eight animals of each avian species (4♂/4♀). All birds were considered to be clinically healthy based on physical examination prior and during the experiment, macroscopic examination at necropsy after the experiment (except AGP), and regular clinical testing of the AGP (including physical examinations, haematology and biochemistry, and polymerase chain reaction analyses for the presence of psittacine circovirus, bornavirus and *Chlamydia psittaci*). All animals were group housed per species, except the AGP (singly housed in 0.9 m by 0.9 m by 0.7 m cages), and had *ad libitum* access to commercial feed and drinking water. The light schedule was adapted to 18 hours of light and 6 hours of darkness. The temperature was monitored and kept between 18 and 25 °C, the relative humidity was between 40 and 80%.

### Experimental design

All animals, except the AGP, received iohexol and creatinine by cannulating the *vena cutanea ulnaris superfacialis* (wing vein) with a 25-gauge IV catheter (Terumo versatus winged IV catheter, Terumo Europe, Leuven, Belgium). The parrots were first anaesthetized for 5 min with isoflurane (IsoFlo, Abbott Laboratories LTD, Madrid, Spain) and oxygen inhalation, followed by catheterization of the *vena metatarsalis plantaris superficialis* (leg vein). After 10 min following recovery from anaesthesia, IV administration to the AGP was then performed via a 26-gauge catheter (Terumo Europe). IV administration was performed as follows. First, a bolus of 64.7 mg/kg BW (0.1 mL/kg BW) Omnipaque® 300 mg I/mL (GE, Healthcare, Belgium) was administered to all species, except pigeons and AGP. Before administratin to the pigeons and the AGP, the Omnipaque® solution was diluted four times (1:4) in 0.9% sodium chloride (NaCl). After administration, the IV catheters were flushed with an equal volume of 0.9% NaCl. Second, an IV injection of 40 mg/kg BW creatinine hydrochloride in 0.9% NaCl solution (Sigma Aldrich, Bornem, Belgium) was immediately administered after iohexol to all species and the catheters were flushed according to the iohexol administration. Blood samples (0.5 mL in chickens, turkeys, ducks and 0.3 mL in pigeons) of all birds, except the parrots, were collected from the *vena metatarsalis plantaris superficialis* by direct venipuncture (25 G needle) and drawn into heparin collection tubes at 0 (before administration) and 5, 15, 30, 60, 120, 180, 360, 480 and 600 min post administration (p.a.). Blood sampling of the AGP (0.3 mL) was first performed through the catheter in the metatarsal vein (0, 5, 15, 30 and 60 min). After 60 min this catheter was removed, and subsequently, blood was withdrawn from the *vena jugularis* by direct venipuncture (30 G needle; 120, 180, 360, 480 and 600 min p.a.). Blood samples were transferred on ice and centrifuged for 10 min (2,851 rpm, 4 °C) within two hours after blood collection. Plasma was aliquoted and stored at −20 °C until analysis. After euthanasia of the birds (except the parrots) with an overdose of pentobarbital (Sodium pentobarbital 20%®, Kela, Hoogstraten, Belgium), both kidneys were removed, dry-dipped and weighted to determine the KW.

### Laboratory analysis

Exo-iohexol concentrations were determined by a validated high-performance liquid chromatography method with ultraviolet detection (HPLC-UV)^32^. The method was fully validated in broiler chicken plasma, followed by a more brief validation in the other avian species. Detailed method validation results can be found in Supplementary Material S1. Briefly, 25 µL of the internal standard iohexol impurity J (chemical reference substance of the European Pharmacopoeia, Strasbourg, France) were added to 100 µL (chicken, turkey, duck) or 50 µL (pigeon, AGP) of plasma, followed by a deproteinization with trifluoroacetic acid (Sigma Aldrich) and a centrifugation step (13,000 rpm, 10 min, 4 °C). Exo-iohexol concentrations were assayed after injecting 50 µL (chicken, turkey, duck) or 20 µL (pigeon, AGP) of the supernatant onto the HPLC-UV instrument. The LOQ of exo-iohexol (µg/mL) was as follows: chicken, turkey, duck: 0.46; pigeon, AGP: 0.91. Creatinine concentrations were determined by an in-house validated enzymatic method, as described by Van Hoek *et al*.^18^, using a Catalyst Dx® Chemistry Analyser (Idexx Laboratories, Westbrook, USA). The upper and lower detection limit was 1.02 and 135.97 µg/mL, respectively. The method was validated in turkey plasma, followed by a more brief validation in the other avian species. Detailed method validation results can be found in Supplementary Material S2. Plasma concentration-time curves were constructed, and the area under the plasma concentration-time curve (AUC_0->t_) of each clearance marker was computed by the trapezoidal rule with extrapolation to infinity using non-compartmental pharmacokinetic analysis (WinNonlin® 6.3, Pharsight, Missouri, USA). Plasma clearance of exo-iohexol and creatinine were calculated by dividing the dose by the AUC_0->t_. Allometric scaling was applied on the clearance using BW and KW^33^.

### Statistical analysis

The plasma clearances of both markers were compared between avian species (within one technique) and between techniques (within one bird species). Within each technique, the effect of bird species was analysed using one-way analysis of variance (ANOVA, SPSS 24.0, IBM, USA). Within each bird species, the effect of technique was further analysed in a mixed model with repeated measurements on technique. The effect of technique was compared between the different sexes. Post hoc comparisons test was performed according to Scheffé. The level of significance was set at 0.05. The correlation among BW and KW was quantified by the Spearman rank correlation coefficient.

## Supplementary information

Comparative physiology of glomerular filtration rate by plasma clearance of exogenous creatinine and exo-iohexol in six different avian species

## Data Availability

The datasets generated during and/or analysed during the current study are available from the corresponding author on reasonable request.
