# Supplementary material for: Comparative physiology of glomerular filtration rate by plasma clearance of exogenous creatinine and exo-iohexol in six different avian species
Source: Sci Rep. 2019 Dec 23;9:19699. doi: 10.1038/s41598-019-56096-5 (PMC6928228; doi:10.1038/s41598-019-56096-5)
Supplement: Supplementary file 1 — Comparative physiology of glomerular filtration rate by plasma clearance of exogenous creatinine and exo-iohexol in six different avian species [file 41598_2019_56096_MOESM1_ESM.docx]

**Comparative physiology of glomerular filtration rate by plasma clearance of exogenous creatinine and exo-iohexol in six different avian species**

Elke Gasthuys, Andres Montestinos, Nele Caekebeke, Mathias Devreese, Siegrid De Baere, Maria Ardiaca, Dominique Paepe, Siska Croubels, Gunther Antonissen

**Supplementary Materials**

**S1. Method validation results of the HPLC-UV method for exo-iohexol analysis**

The following method validation parameters were determined: linearity, within- and between-run precision and accuracy, limit of detection (LOD) and limit of quantification (LOQ).

1. *Linearity*

A calibration curve (0.5 – 750 µg/mL) was prepared by spiking blank plasma samples. The correlation coefficient (R, all species > 0.998) and goodness-of-fit coefficient (g, all species between 2.02 and 9.63%) were assessed. The acceptance criteria were defined as ≥ 0.99 and ≤ 10%, respectively.

1. *Within- and between-run precision and accuracy*

Six blank plasma samples were spiked with iohexol at low and high concentration levels (10 and 100 µg/mL, respectively), on the same day (within-run) and on three different days (between-run). The acceptance criteria were defined as -20% to 10% of the theoretical concentration. The precision was defined as the relative standard deviation (RSD): RSD_max_ = 2^(1-0.5logConc)^ x 2/3 (within-run precision); RSD_max_ = 2^(1-0.5logConc)^ (between-run precision). The acceptability criteria were met in all avian species at the predefined levels for exo-iohexol.

1. *Limit of detection (LOD)/quantification (LOQ)*

The LOQ was defined as the lowest concentration of exo-iohexol which could be quantified with a precision and accuracy that fell within the acceptability criteria. The LOD was the lowest concentration which could be recognized by the detector with a signal-to-noise ratio of ≥ 3. The LOD and LOQ values for exo- iohexol in all six avian species are shown in **Table S1**.

**Table S1.** Limit of detection (LOD), Limit of quantification (LOQ) for exo- iohexol in six avian species.

| **Species** | **LOD**  **(x10^-3^ µg/mL)** | **LOQ**  **(µg/mL)** |
| --- | --- | --- |
| Broiler chicken exo | 0.81 | 0.46 |
| Laying chicken exo | 7.08 | 0.46 |
| Turkey exo | 0.38 | 0.46 |
| Pigeon exo | 0.07 | 0.91 |
| Duck exo | 0.81 | 0.46 |
| Parrot exo | 2.45 | 0.91 |

**S2. Method validation results of the enzymatic creatinine assay**

The following method validation parameters were determined: linearity, within- and between-run precision and accuracy, and limit of quantification (LOQ).

1. *Linearity*

A calibration curve (1.0 – 100 µg/mL) was prepared by spiking blank plasma samples. The correlation coefficient (R, all species > 0.998) and goodness-of-fit coefficient (g, all species between 2.02 and 9.63%) were assessed. The acceptance criteria were defined as ≥ 0.99 and ≤ 10%, respectively.

1. *Within- and between-run precision and accuracy*

Six blank plasma samples were spiked with creatinine at low and high concentration levels (10 and 50 µg/mL, respectively), on the same day (within-run) and on three different days (between-run). The acceptance criteria were defined as -20% to 10% of the theoretical concentration. The precision was defined as the relative standard deviation (RSD): RSD_max_ = 2^(1-0.5logConc)^ x 2/3 (within-run precision); RSD_max_ = 2^(1-0.5logConc)^ (between-run precision). The acceptability criteria were met at the predefined levels for creatinine.

1. *Limit of quantification (LOQ)*

The LOQ was initially defined by the lowest concentration of creatinine found by Scope et al. (2013) (= 2 µg/mL) and in-house confirmed by spiking six blank samples with creatinine at the 2 µg/mL concentration.
